# Multi-Omics Investigation into Why Viable Oogonial Stem Cells Can Still Be Isolated and Cultured from Post-Mortem *Paralichthys olivaceus*

**DOI:** 10.3390/ijms262110679

**Published:** 2025-11-02

**Authors:** Yuqin Ren, Yucong Yang, Nuan He, Guixing Wang, Zhongwei He, Yufeng Liu, Wei Cao, Xiaoyan Zhang, Yitong Zhang, Lize San, Zengsheng Han, Jilun Hou

**Affiliations:** 1 State Key Laboratory of Mariculture Biobreeding and Sustainable Goods, Beidaihe Central Experiment Station, Chinese Academy of Fishery Sciences, Qinhuangdao 066100, China; renyq@bces.ac.cn (Y.R.); yangyc@bces.ac.cn (Y.Y.); 15226588061@163.com (N.H.); wanggx@bces.ac.cn (G.W.); hezw@bces.ac.cn (Z.H.); liuyf@bces.ac.cn (Y.L.); caow@bces.ac.cn (W.C.); zhangxy@bces.ac.cn (X.Z.); zhangyt@bces.ac.cn (Y.Z.); sanlz@bces.ac.cn (L.S.); 2 Hebei Key Laboratory of the Bohai Sea Fish Germplasm Resources Conservation and Utilization, Beidaihe Central Experiment Station, Chinese Academy of Fishery Sciences, Qinhuangdao 066100, China; 3 Bohai Sea Fishery Research Center, Chinese Academy of Fishery Sciences, Qinhuangdao 066100, China; 4 Hebei Key Laboratory of Nano-Biotechnology, Hebei Key Laboratory of Applied Chemistry, College of Environmental and Chemical Engineering, Yanshan University, Qinhuangdao 066004, China

**Keywords:** germ stem cells, *Paralichthys olivaceus*, post-mortem interval, transcriptomics, metabolomics, oxidative stress

## Abstract

The cryopreservation and transplantation of germline stem cells (GSCs) have become the key to conserving fish genetic resources and safeguarding species diversity. This study aimed to investigate the effects of post-mortem temperature and time on the preservation of oogonial stem cells (OSCs) in the marine fish *Paralichthys olivaceus*. OSCs remained viable after fish death, and they remained viable and could be cultured after storage at 19 °C for 15 h and at 4 °C for 24 h. Combined transcriptomic and metabolomic analysis was used to identify the pathways leading to OSC death. Several genes were differentially expressed in the ovarian tissue post-mortem, with the most enriched pathways being ferroptosis, fatty acid metabolism/biosynthesis, glutathione metabolism, citric acid cycle (TCA cycle), and arachidonic acid metabolism signaling pathways. Genes related to ferroptosis, such as *vdac2*, *p53*, and *slc7a11*, as well as metabolites such as adrenic acid and arachidic acid, can serve as reliable biomarkers for evaluating the viability of post-mortem OSCs. These findings provide valuable insights and theoretical support for the effective use of post-mortem GSCs and enhance strategies for germplasm resource conservation in fish.

## 1. Introduction

Fish biodiversity is decreasing owing to overfishing, pollution, and climate change. Therefore, conserving significant genetic resources and maintaining long-term protection is critical. Germplasm resources are conserved in vivo by establishing protected areas, breeding farms, and ex situ conservation facilities. However, this approach faces several challenges for endangered fish species, including high costs, slow results, and delayed intervention. Once fish die, their genetic resources of fish species cannot be passed on to future generations [1,2]. The in vitro preservation of germplasm includes the preservation of cell resources, such as sperm, oocytes, embryos, somatic cells, and stem cells, etc., and isolated chromosomes, DNA, and other genetic resources [3]. Fish germ stem cells (GSCs) have advantages over eggs and embryos owing to their developmental plasticity and bidirectional differentiation, which are essential for maintaining genetic continuity [4,5]. The transplantation of GSCs to conserve genetic resources has been successfully implemented in the rainbow trout (*Oncorhynchus mykiss*), Chinese sturgeon (*Acipenser sinensis*), and tiger puffer (*Takifugu rubripes*) and is of significant importance for biodiversity and the protection of endangered genetic resources [6,7,8,9].

GSCs for transplantation are commonly isolated fresh from gonadal tissues [10,11] or from frozen gonadal tissues [12,13]. However, isolating and conserving GSCs in wild and endangered fish remains difficult when death occurs under uncontrolled circumstances and cell isolation takes time [7]. Fish are often stored at an ambient temperature for several hours or days after death, with or without refrigeration [14]. During this period, tissue breakdown complicates the collection of viable cells and creates a considerable barrier to germplasm conservation [15].

Post-mortem cell isolation and its applications have mainly been studied in humans, with the harvesting of stem cells from cadaveric tissues emerging as a promising regenerative medicine approach [16,17,18,19]. Post-mortem adipose-derived mesenchymal stromal/stem cells can be readily isolated and cultured from the adipose tissue [16]. Viable and functional hepatocytes can be isolated within 12 h of thermal ischemic death [17], and transplantable hepatic cells can be isolated within 27 h of storage at 4 °C [18]. Additionally, mesenchymal stem cells have been isolated from equine cadaver suspensory ligaments within 48–72 h post-mortem [19]. Post-mortem gene expression studies in humans aimed to identify reliable molecular markers to improve the prediction of the time of death [20] and models have been developed to predict the time of death based on the effects of post-mortem cold ischemia on tissue transcriptomes [21]. Recent studies have demonstrated the usefulness of RNA expression analysis for post-mortem interval estimation [22,23,24]. To our knowledge, no prior reports have documented successful isolation and culture of GSCs from fish after death, or on post-mortem gene expression and metabolite changes in fish.

Here, we investigated the effects of post-mortem temperature and time on the preservation of oogonial stem cells (OSCs) in a marine fish, *Paralichthys olivaceus*. We aimed to determine the critical window of time for optimal OSC preservation by collecting and culturing the gonadal tissue of *P. olivaceus* that had been stored post-mortem at either 4 °C or 19 °C. Additionally, transcriptomic and metabolomic analyses were used to determine metabolite profiles, differential gene expression, and key genes and metabolites that could be used as biomarkers for evaluating the viability of post-mortem OGCs. These findings provide valuable insights and theoretical support for the effective use of post-mortem GSCs and enhance strategies for germplasm resource conservation in fish

## 2. Results

### 2.1. In Vitro Culture of OSCs at Different Time Intervals After Death

**Figure 1:**
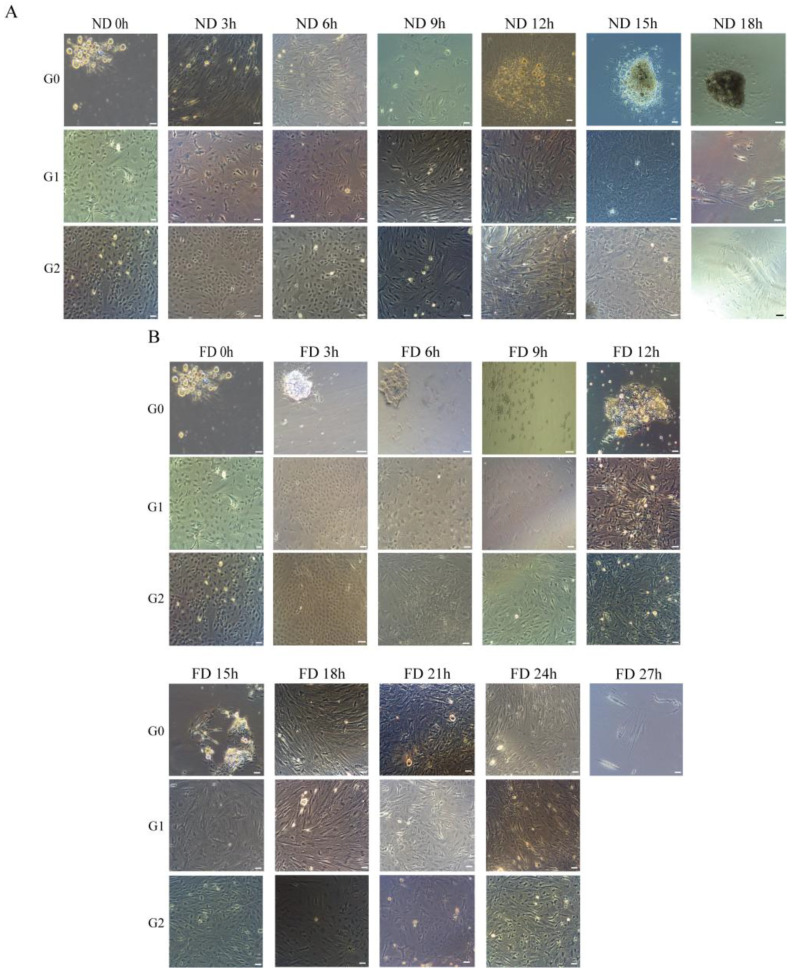
Morphology of *P. olivaceus* oogonial stem cells established from post-mortem ovarian tissues at different time intervals. (**A**) ND group (stored at 19 °C). (**B**) FD group (stored at 4 °C). The scale bar in all panels represents 50 µm.

We examined the in vitro culture of cells from ovarian tissues harvested at different post-mortem intervals. The results from the ND (Normal Digestion temperature) group showed that OSCs could be passaged to at least the third generation when isolated within 15 h post-mortem at 19 °C (ND0h–ND15h), but not at ND18h (Table A1). Similarly, in the FD (Frozen Digestion temperature) group, OSCs could be passaged to at least the third generation when isolated within 24 h post-mortem at 4 °C (FD0h–FD24h), but not at FD27h (Table A2). Morphological examination revealed that ND15h cells migrated from the tissue explant, and the morphology of the passaged cells was comparable to that of the 0 h control (Figure 1A). However, attempts to establish cell lines at ND18h were unsuccessful, as cells initially adhered but subsequently enlarged and underwent apoptosis over time (Figure 1A).

As shown in Figure 1B, FD24h cells adhered to the culture surface, and the passaged cells displayed irregular polygonal morphologies. In contrast, FD27h cultures contained only sparse adherent cells, which exhibited an enlarged, flattened morphology and ultimately lost viability (Figure 1B). Notably, cell lines isolated and cultured separately from both ND15h and FD24h have been maintained for over 50 passages.

**Figure 2:**
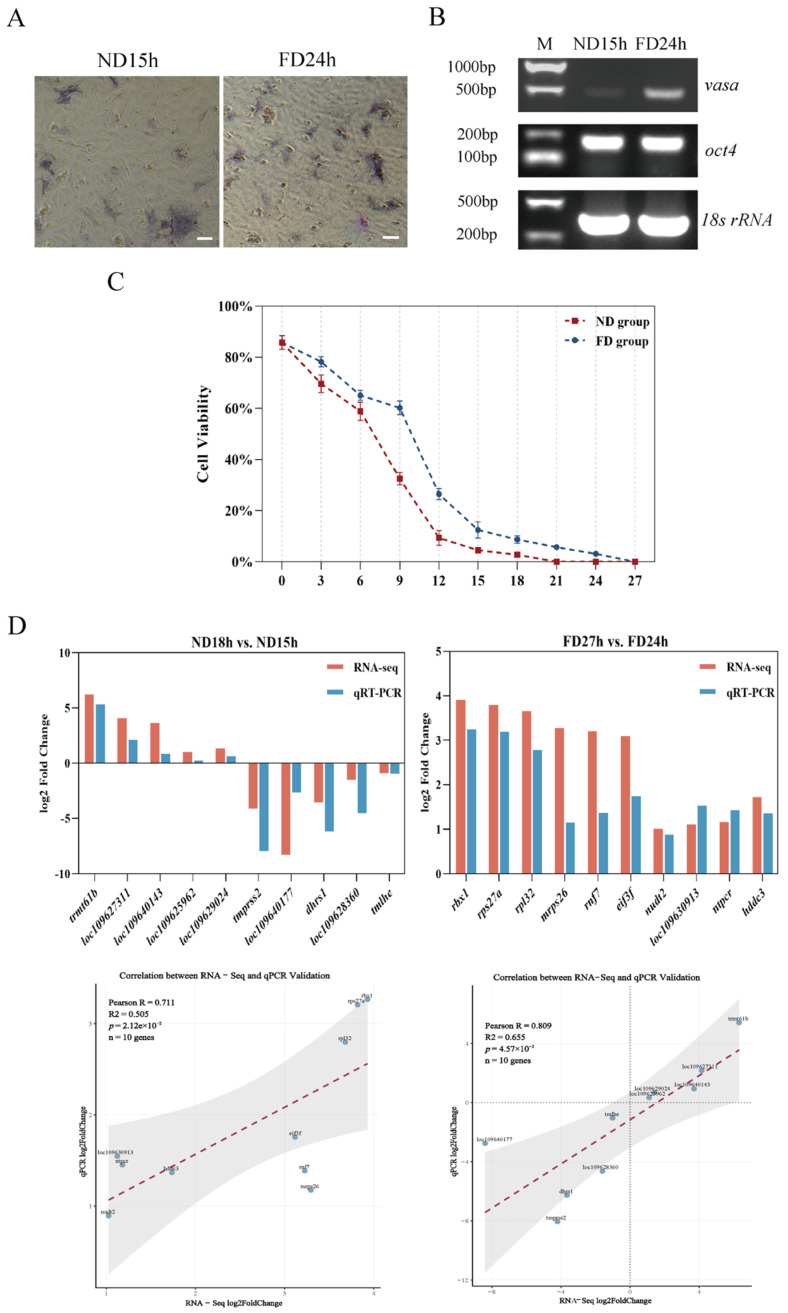
(**A**) Alkaline phosphatase detects cell stemness. Scale bar: 50 µm. (**B**) Marker genes identify cell tissue type and stemness expression. M: maker; *vasa*: 502 bp; *oct4*: 205 bp; 18s rRNA: 257 bp. (**C**) Cell viability assessment by trypan blue exclusion. Data represent mean ± SD of three independent biological replicates (*n* = 3), with approximately 500 cells counted per replicate. ND group: red line; FD group: blue line. (**D**) Validation of RNA-Seq results by qRT-PCR. Comparison of log2FoldChange values between RNA-Seq (x-axis) and qRT-PCR (y-axis) for 10 selected genes (*n* = 3). Pearson correlation coefficient (R) and *p*-value are shown.

**Figure A1:**
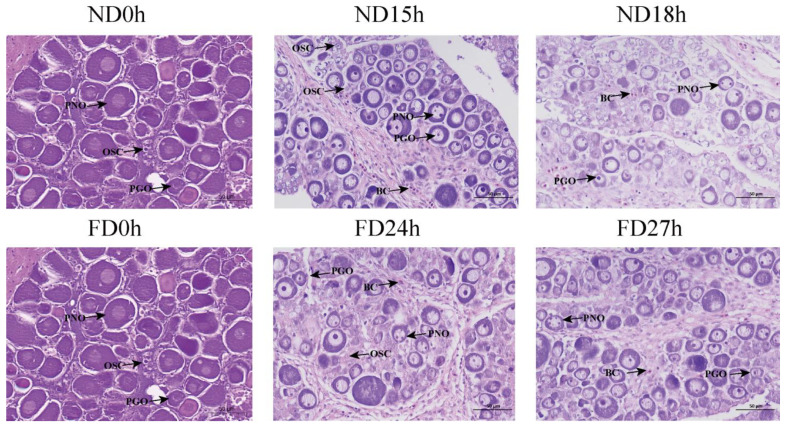
The histology of ovarian tissues at ND0h, ND15h, ND18h, FD0h, FD24h, and FD27h. OSC: oogonial stem cell; PGO: primary growth oocytes; PNO: perinucleolar oocyte; BC, blood cells; Scale: 50 μm.

This loss of culturability was directly correlated with a progressive decline in cell viability, as assessed by trypan blue staining. The FD group maintained a cell survival rate above 60% for 9 h, whereas the ND group maintained a rate above 50% for 6 h. Survival rates decreased progressively in both groups, with the ND group exhibiting significantly lower viability than the FD group. The ND group showed complete loss of viability by 21 h, while the FD group maintained viability until 27 h (Figure 2C). Observation of histological sections showed preserved oocyte morphology with no significant signs of cellular degradation or structural damage in ovarian tissues across all designated time points (0h, ND15h, ND18h, FD24h, and FD27h) (Figure A1). We also observed that the gonadal index (GSI) was higher at ND18h and lower at ND15h, indicating that cell viability or culture success was not affected by GSI (Table A9).

### 2.2. Detection of Stem Cells Using Alkaline Phosphatase and RT-PCR

Staining for alkaline phosphatase showed strong intracellular purplish-red precipitate in the majority of cells, confirming the presence of oogonial stem cells in the ND15h and FD24h groups (Figure 2A). Further gene expression analysis showed that the germ stem cell marker genes *vasa*, *oct4* and the cell origin gene 18s rRNA from *P. olivaceus* expressed in the cell lines (Figure 2B). *Vasa* is a highly conserved RNA helicase specifically expressed in germline cells across animal species, serving as a definitive marker for the germ cell lineage [25]. *Oct4* (also known as *pou5f1*), a POU-domain transcription factor, is a critical regulator of pluripotency and self-renewal in stem cells [26]. These results indicated that the cell line is oogonia stem cells.

### 2.3. Identification of DEGs at Different Times of ND Group and FD Group

To investigate the molecular mechanisms underlying the observed decline in cell viability and loss of culturability, we performed transcriptomic analysis on ovarian tissues by comparing ND18h vs. ND15h (where OSC cultures failed versus succeeded at 19 °C) and FD27h vs. FD24h (where OSC cultures failed versus succeeded at 4 °C). High-quality transcriptomes were obtained from three independent biological replicates per time point (Q20 > 97%; mapping rate > 90%; Table A3 and Table A4), yielding 21,087 expressed genes. Differential expression analysis was performed by comparing the last viable time point with the first non-viable time point within each storage condition (ND18h vs. ND15h and FD27h vs. FD24h). This comparison identified 91 differentially expressed genes (DEGs) (48 upregulated and 43 downregulated) in the ND group and 3887 DEGs (1942 upregulated and 1945 downregulated) in the FD group. The reliability of the transcriptome data was confirmed by qRT-PCR (Figure 2D).

**Figure 3:**
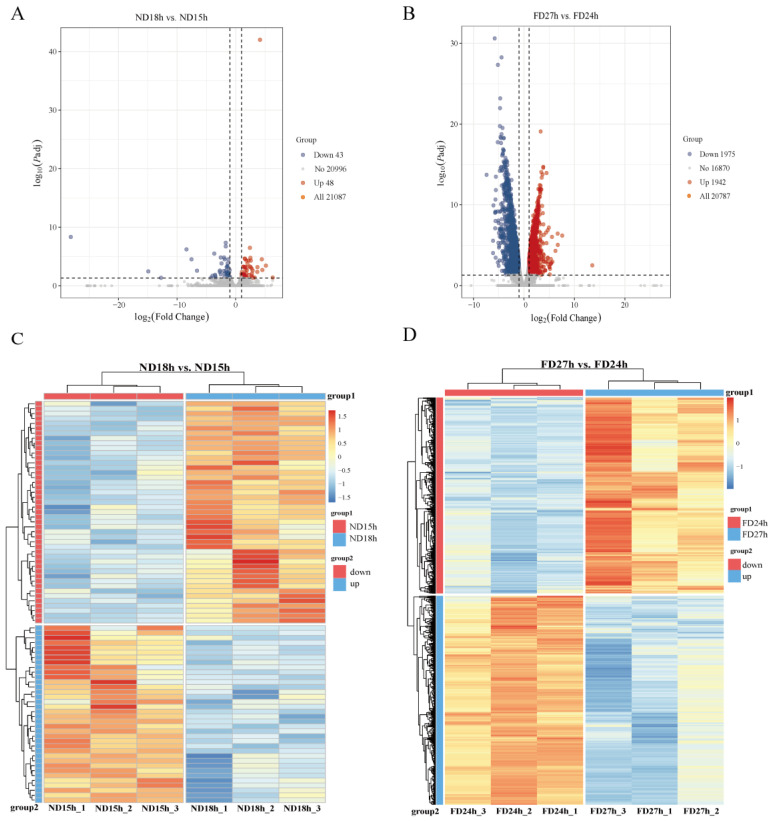
(**A**,**B**) Volcano plots of differentially expressed genes (DEGs) with thresholds of |log2FoldChange| > 1 and padj < 0.05. Red points: significantly upregulated genes; blue points: significantly downregulated genes; gray points: non-significant genes (*n* = 3). (**C**,**D**) Hierarchical clustering heatmaps of DEGs. Color scale represents Z-score normalized expression values.

Subsequently, heat maps were generated based on the differences in gene expression patterns, and the six samples clustered distinctly between ND and FD groups (Figure 3C,D).

### 2.4. Enrichment Analysis of DEGs and Non DEGs

**Figure 4:**
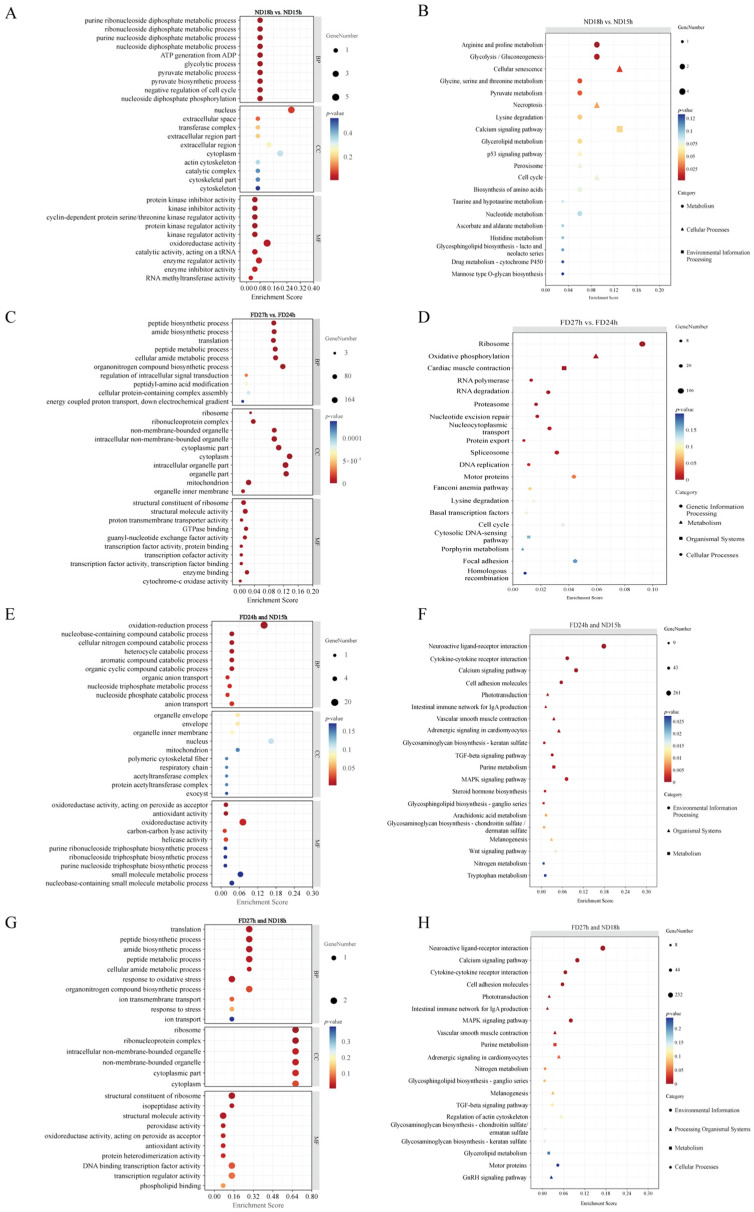
GO enrichment analysis and KEGG pathway enrichment analysis in ND18 vs. ND15h DEGs (**A**,**B**), FD27 vs. FD24h DEGs (**C**,**D**), FD24h and ND15h non DEGs (**E**,**F**) and FD27h and ND18h non DEGs (**G**,**H**) (*n* = 3). GO enrichment analysis of DEGs and non-DEGs. Bar length represents −log10 (*p*-value). Only significantly enriched terms (*p* < 0.05) are shown. KEGG pathway enrichment analysis. Bubble size represents gene count; color represents −log10 (*p*-value).

To better understand the biological significance of DEGs between ND18h vs. ND15h, GO enrichment analysis identified 91 DEGs that were significantly associated with biological processes such as pyruvate metabolism, glycolysis, nucleoside diphosphate phosphorylation, ATP generation, and nucleoside diphosphate metabolism. Molecular functions are linked to protein kinase inhibitors and related regulatory activities. (Figure 4A). The top 20 enriched pathways showed that these DEGs were mainly involved in cellular processes such as senescence, necroptosis, p53 signaling, peroxisome function, cell cycle, calcium signaling in environmental processing, and various metabolic pathways, including arginine and proline metabolism, glycolysis/gluconeogenesis, and pyruvate metabolism (Figure 4E and Table A5). These findings suggest that the failure of OSC culture at 19 °C beyond 15 h is associated with disruption in energy metabolism and activation of specific cell death pathways.

The 3887 DEGs in FD27h vs. FD24h were significantly enriched in various GO categories, including peptide and amide biosynthetic and metabolic processes in biological processes; ribosome structure, molecule activity, and transcription factor activity in molecular functions; and ribosome and ribonucleoprotein complexes in cellular components (Figure 4B). The top 20 pathway maps indicated the involvement of ribosomes, RNA polymerase, RNA degradation, and proteasomes in genetic information processing; oxidative phosphorylation, lysine degradation, and steroid biosynthesis in metabolism; and cardiac muscle contraction in organismal systems (Figure 4F and Table A6). This comprehensive disruption of core cellular machinery, particularly in protein synthesis and energy production, underlies the loss of cell viability under refrigerated conditions beyond 24 h.

For the 6593 non DEGs (padj > 0.05, log fold change ± 0.5) in FD24h and ND15h, GO enrichment analysis revealed significant involvement in ion transport, immune response, immune system process, developmental process, and anatomical structure development within biological processes. In terms of molecular functions, they were linked to G protein-coupled receptor activity, channel activity, and passive transmembrane transporter activity, whereas in cellular components, they were associated with the extracellular region (Figure 4C). The top 20 enriched pathways showed that these non-DEGs were primarily involved in neuroactive ligand-receptor interactions, cytokine-cytokine receptor interactions, calcium signaling pathways, and MAPK signaling pathways in environmental information processing, as well as in purine metabolism (Figure 4G and Table A7). The stability of these genes and pathways in culturable cells suggests their essential role in maintaining fundamental cellular functions necessary for viability.

For the 6081 non DEGs in FD27h and ND18h, GO enrichment analysis revealed significant involvement in ion transport, immune response, cell adhesion, and biological adhesion within biological processes; G-protein coupled receptor activity, channel activity, and passive transmembrane transporter activity in molecular functions; and the extracellular region and matrix in cellular components (Figure 4D). The top 20 enriched pathways showed that these non-DEGs were primarily associated with neuroactive ligand-receptor interactions, calcium signaling, cytokine-cytokine receptor interactions, and MAPK signaling pathways in environmental information processing, as well as purine metabolism (Figure 4H and Table A8). The preservation of these core cellular processes even in non-viable cells indicates they represent fundamental cellular infrastructure that remains intact despite the activation of cell death mechanisms.

### 2.5. Identification of DEMs at Different Times of ND Group and FD Group

**Figure 5:**
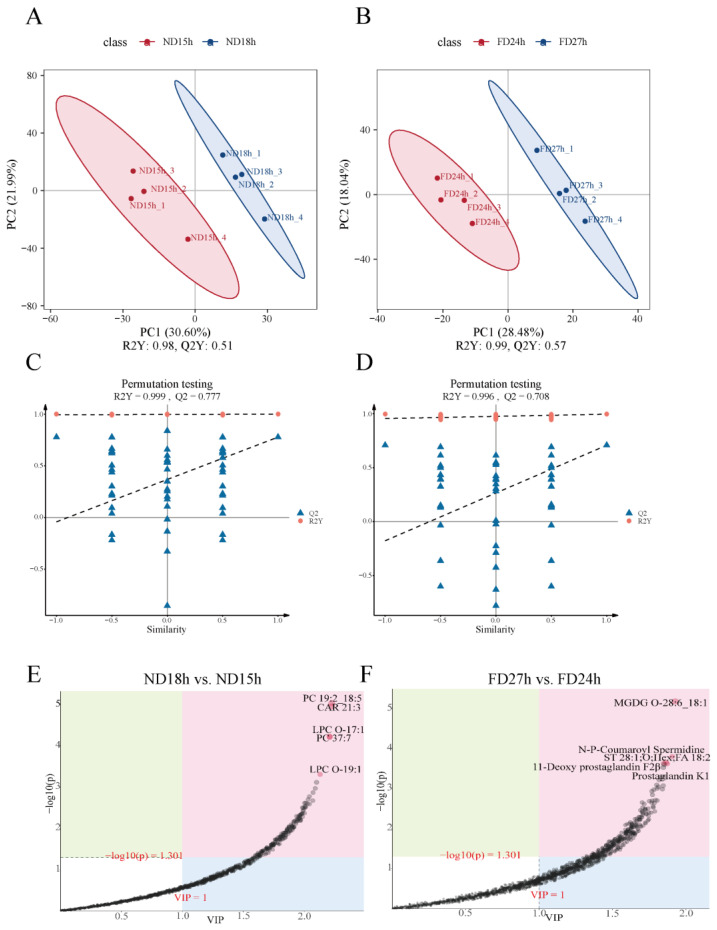
Score plots for PLS-DA in the (**A**) ND group and (**B**) FD group (*n* = 4). Permutation Test Plot and feature importance metabolites for OPLS-DA in the (**C**,**E**) ND18h vs. ND15h and (**D**,**F**) FD27h vs. FD24h. Notes: In the score plots, the closer R2Y and Q2 are to 1, the more stable and reliable the model is, Q2 > 0.5 indicates stable and reliable model, 0.3 < Q2 ≤ 0.5 indicates moderate model stability and reliability, and Q2 < 0.3 indicates poor model stability and reliability. In the loading plot, the variables are farther away from the origin in the direction of the horizontal coordinate contributes more to the differentiation between the two sets of samples.

Metabolite profiling was performed using four independent biological replicates per time point. The PLS-DA score plot showed that samples from both the ND and FD groups were clearly differentiated in the principal components (Figure 5A,B). The evaluation parameters obtained from OPLS-DA are (ND18h vs. ND15h (R^2^Y = 0.999 cumulative, Q^2^ = 0.777 cumulative) and FD27h vs. FD24h (R^2^Y = 0.996 cumulative, Q^2^ = 0.708), indicating stable and reliable results (Figure 5C,D). The loading plot results revealed that the metabolites with the greatest contribution to the ND18h vs. ND15h groups were predominantly phospholipids, including PC 19:2_18:5, CAR 21:3, LPC O-17:1, PC 37:7, and LPC O-19:1 (Figure 5E). In contrast, the metabolites with the greatest contribution in the FD24h vs. FD27h groups were primarily glycolipids, such as MGDG O-28:6_18:1, N-P-Coumaroyl Spermidine, ST 28:1;O;Hex;FA 18:2, 11-Deoxy prostaglandin F2β, and Prostaglandin K1 (Figure 5F). These metabolites play a key role in the structural formation of cell membranes, helping maintain membrane stability and fluidity.

### 2.6. Enrichment Analysis of DEMs

**Figure 6:**
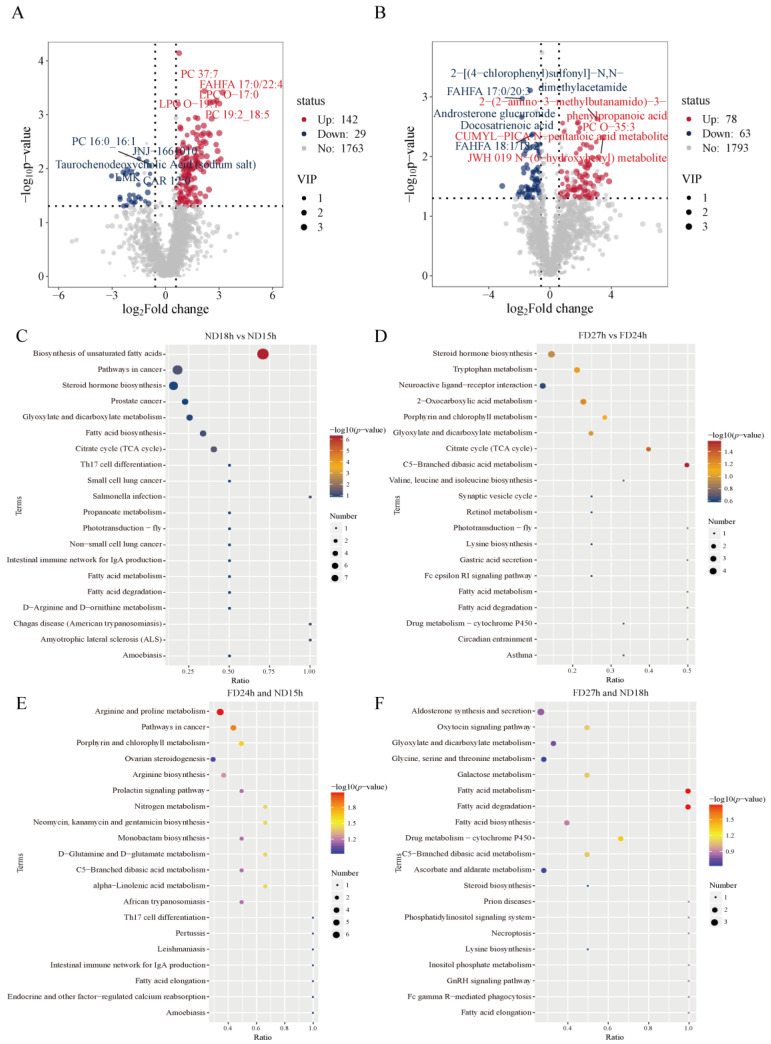
(**A**,**B**) Volcano plot analysis and (**C**,**D**) KEGG pathway enrichment analysis of differentially expressed metabolomics in ND18h vs. ND15h and FD27h vs. FD24h (*n* = 4). KEGG pathway enrichment analysis of (**E**) FD24h and ND15h, (**F**) FD27h and ND18h non DEMs (*n* = 4).

Of the 171 DEMs selected in the ND18h versus ND15h group, PC 19:2_18:5, PC 37:7, LPC O-19:1, LPC O-17:0, and FAHFA 17:0/22:4 were significantly upregulated. JNJ-1661010, PC 16:0_16:1, Taurochenodeoxycholic Acid (sodium salt), LMK, and CAR 12:0 were significantly down-regulated (Figure 6A). Among the 146 DEMs selected in the FD24h vs. FD27h group, 2-(2-amino-3-methylbutanamido)-3-phenylpropanoic acid, PC O-35:3, JWH 019 N-(6-hydroxyhexyl) metabolite, N-(6-hydroxyhexyl) metabolite and CUMYL-PICA N-pentanoic acid metabolite were significantly up-regulated, 2-[(4-chlorophenyl)sulfonyl]-N,N-dimethylacetamide, FAHFA 17:0/20:3, Androsterone glucuronide, Docosatrienoic acid and FAHFA 18:1/18:2 were significantly down-regulated (Figure 6B). Among the top 10 enriched pathways between the ND15h and ND18h DEMs, the Biosynthesis of unsaturated fatty acids, glyoxylate and dicarboxylate metabolism, citrate cycle (TCA cycle), and fatty acid biosynthesis were significant (Figure 6C). Among the top 10 enriched pathways between the FD24h and FD27h DEMs, 2-Oxocarboxylic acid metabolism, citrate cycle (TCA cycle), porphyrin and chlorophyll metabolism, and C5-Branched dibasic acid metabolism were noticeable (Figure 6D). Arginine and Proline Metabolism and Porphyrin and Chlorophyll Metabolism were significantly enriched in FD24h and ND15h non-DEMs (Figure 6E), whereas Glyoxylate and Dicarboxylate Metabolism, Glycine, Serine and Threonine Metabolism, and Fatty Acid Metabolism/Degradation were enriched in FD27h and ND18h non-DEMs (Figure 6F).

### 2.7. Joint Analysis of Transcriptome and Metabolomics

**Figure 7:**
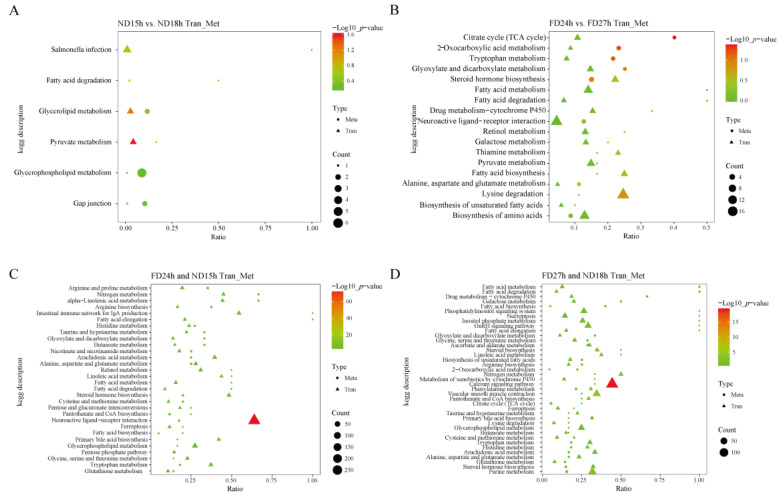
(**A**–**D**) The shared pathways enriched by the com bined analysis of the transcriptome and metabolome.

To gain a systems-level understanding of the molecular events leading to the loss of cell viability, we performed an integrated analysis of the transcriptomic and metabolomic data from the ND and FD groups. Pathway analysis revealed distinct metabolic disruptions associated with the failure of cell culture in each group. In the ND group, the transition from culturable (ND15h) to non-culturable (ND18h) states was characterized by significant alterations in six key pathways, including fatty acid degradation, glycerolipid metabolism, and pyruvate metabolism, indicating a severe disruption in energy homeostasis and membrane integrity (Figure 7A). A more extensive metabolic collapse was observed in the FD group during the critical window between FD24h and FD27h, involving 18 pathways such as the TCA cycle, fatty acid metabolism, and amino acid biosynthesis, reflecting a systemic failure of core metabolic processes (Figure 7B).

Further analysis focused on the molecular signatures distinguishing culturable (CE: FD24h and ND15h) from non-culturable (NCE: FD27h and ND18h) conditions. In the CE group, the stability of 6593 genes and 1212 metabolites was associated with 30 shared pathways crucial for cellular maintenance. These included fatty acid metabolism and elongation, which help preserve membrane stability, alongside the activation of glutathione metabolism and the pentose phosphate pathway, which collectively protect cells from oxidative stress (Figure 7C). Conversely, the NCE group, characterized by 6081 stable genes and 1376 stable metabolites, exhibited 39 shared pathways predominantly linked to cell death processes. These were categorized into three major events: (1) activation of specific cell death pathways such as necroptosis and ferroptosis; (2) pathways associated with oxidative stress and mitochondrial dysfunction, including impaired fatty acid degradation and glutathione metabolism; and (3) pathways related to apoptosis, such as perturbations in the TCA cycle and calcium signaling (Figure 7D).

Crucially, a direct comparison between the CE and NCE groups identified arginine and proline metabolism and alpha-linolenic acid metabolism as upregulated in viable cells, while the calcium signaling pathway was downregulated. We propose that these specific pathway differences are critical determinants for the successful in vitro culture of cells, highlighting key metabolic features that distinguish viable OSCs from those that have undergone irreversible damage.

## 3. Discussion

We discovered that viable cells from *P. olivaceus* ovaries could be harvested and successfully cultured (>50 passages) in vitro after preservation at 4 °C for up to 24 h or at 19 °C for up to 15 h post-mortem while maintaining their stemness. Combined with our previously established transplantation protocol in this species [7], which demonstrated that OSCs from fresh tissues can colonize recipient gonads and produce functional gametes, the current work provides a practical solution for obtaining donor cells from unexpectedly deceased individuals. This approach could be particularly valuable for conserving genetic resources from elite broodstock that die prematurely in aquaculture operations or from endangered individuals found dead in natural habitats. However, the maximum isolation time window at other temperatures requires further investigation. Comparable human studies have shown that hepatocytes remain functional after 12 h of warm ischemia [17] and can regenerate liver tissue when extracted up to 27 h post-mortem after storage at 4 °C [18]. Additionally, storing the brain post-mortem at 4 °C for a week, 15 °C for 48 h, or 25 °C for 24 h can generate neural stem/progenitor cells [15]. Storing ovarian tissues at 4 °C rather than room temperature is essential for preserving their integrity and oocyte viability in both chickens and post-mortem mice [27,28]. This low-temperature storage maintains oocyte quality and quantity and improves in vitro culture success [29]. It slows metabolic processes and tissue autolysis and reduces cellular damage during ischemia. Although this method is widely used for organ preservation [14,30,31], one study found that ovarian tissues stored at low temperatures could delay cell death and have an advantage in cell culture, especially with long-term storage. Similar results have been reported for fibroblasts from animal ear skin [32,33,34]. This indicates the existence of specificity among different species and tissues. While the present study primarily focuses on OSCs, future investigations on spermatogonial stem cells (SSCs) from post-mortem testicular tissues would provide comprehensive support for germplasm resource conservation strategies.

### 3.1. Post-Mortem Energy Metabolism Disruption and Its Impact on Cells

Following death, cessation of blood circulation causes hypoxia, leading to impaired aerobic respiration and increased reactive oxygen species (ROS) production [35,36,37]. We found that genes related to early and middle glycolysis, such as *aldob*, *tpi1*, and *gapdh*, were upregulated. Glycolysis produces pyruvate, which is converted to oxaloacetate by pyruvate carboxylase (PC) and enters the TCA cycle [38]. However, PC expression was significantly downregulated at 27 h post-mortem, similar to eHHADH, which affected fatty acid breakdown and acetyl-CoA production. This indicates a decrease in aerobic metabolism and ATP supply, prompting a shift to anaerobic metabolism and lactate production [39].

Amino acids fuel the TCA cycle and replenish its intermediates, thereby sustaining energy production and metabolic homeostasis [40] while also mediating signaling, antioxidant defense, and stress responses [41]. Post-mortem, we observed widespread disruption of amino acid metabolism, with marked reductions in arginine, tryptophan, alanine, aspartate, glutamate, and lysine levels that collectively compromised the energy supply. Arginine is recognized for its antioxidant capacity in alleviating oxidative stress across multiple species [42]—declining 18 h post-mortem and amplifying ROS accumulation and apoptosis. Alanine, aspartate, and glutamate, which normally sustain proliferation and redox balance via energy provision, nucleotide biosynthesis, and antioxidant defense [43], were also significantly depleted, directly weakening the TCA cycle flux. Meanwhile, elevated L-saccharopine and decreased androsterone glucuronide and 2-hydroxyestradiol further disturb amino acid networks, undermining cellular viability and growth [44].

To sustain short-term survival under the initial post-mortem stress, compensatory mechanisms are activated. Consistent with observations in other fish species such as *Oreochromis niloticus* [45], *Cyprinus carpio* var. *Qingtianensis* [46], *Crucian carp* [47] and *C. auratus* [48], the pentose phosphate pathway (PPP) is activated to generate nucleotide precursors and NADPH for redox maintenance [49]. Our transcriptomic data from P. olivaceus further reveal that sustained hypoxia rapidly suppresses purine metabolism: key genes—including adenylate cyclase 9 (*adcy9*), adenylate cyclase 2 (*adcy2*), phosphoribosylformylglycinamidine synthase (*pfas*), and 5′-nucleotidase, cytosolic II (*nt5c2*) were down-regulated, thereby reducing the ATP and nucleotide pools. This is consistent with observations in pearl gentian grouper under hypoxia, where oxidative phosphorylation and energy production are impaired [39], and aligns with the known conservation of this energy-stress response in vertebrates [50].

Simultaneously, our multi-omics analysis indicates a compensatory activation of the glutathione pathway, in which cysteine-driven GSH synthesis assisted by methionine helps scavenge lipid radicals [51]. However, by 27 h post-mortem in our study, amino acid depletion and continued ROS accumulation overwhelm these defenses, and GSH-mediated repair can no longer restore the redox balance. Consequently, no viable cells can be isolated for in vitro culture, indicating that compensatory antioxidant mechanisms are exhausted and metabolic homeostasis is irreversibly lost.

### 3.2. Post-Mortem Lipid Metabolism Disruption and Its Impact on Cellular Integrity

**Figure 8:**
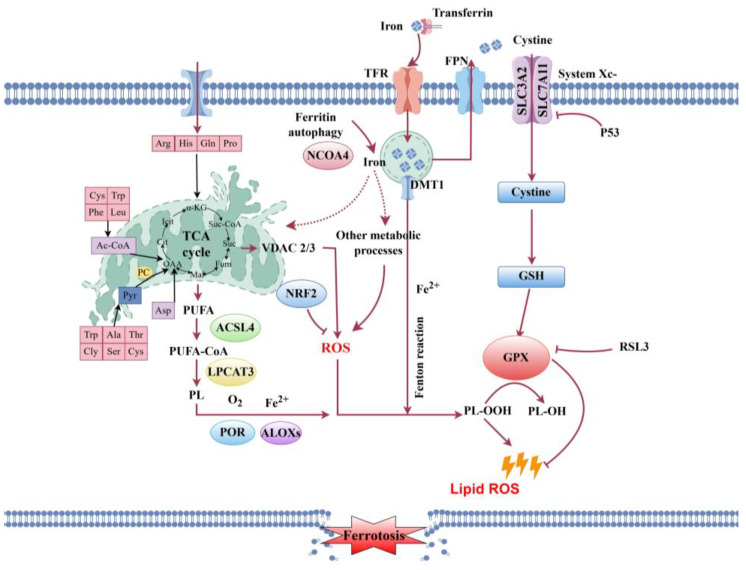
A proposed model illustrating the molecular mechanisms leading to oogonial stem cell death in post-mortem *P. olivaceus* ovarian tissue, based on integrated transcriptomic and metabolomic analyses. The model highlights the central role of oxidative stress and the activation of ferroptosis. Key abbreviations and components are: ROS, reactive oxygen species; TCA cycle, tricarboxylic acid cycle; ACSL4, acyl-CoA synthetase long-chain family member 4; LPCAT3, lysophosphatidylcholine acyltransferase 3; PUFA-PL, polyunsaturated fatty acid-containing phospholipids; PUFA-OOH, peroxidized polyunsaturated fatty acids; SLC7A11, solute carrier family 7 member 11 (a subunit of the system Xc-cystine/glutamate antiporter); GPX4, glutathione peroxidase 4; GSH, reduced glutathione; GSSG, oxidized glutathione. The process occurs within the oogonial stem cell, with key events taking place in the mitochondria (ROS production, TCA cycle dysfunction), the endoplasmic reticulum (involvement of ACSL4 and LPCAT3 in lipid metabolism), and at the plasma membrane (location of the system Xc-antiporter and subsequent lipid peroxidation leading to loss of membrane integrity). In this schematic, black and red arrows indicate promoting or activating relationships, while red arrows with blunt ends indicate inhibitory relationships.

The lipid metabolic network is central to energy and membrane homeostasis and oxidative stress disrupts lipid metabolism [52]. Glycerophospholipids are essential for maintaining membrane structure, organelle function, and cellular energy balance. In this study, PC 40:9 and PC 37:7 were markedly upregulated, providing fluidity and integrity to the lipid bilayer as a compensatory response to membrane damage. In contrast, phospholipases hydrolyze glycerophospholipids into arachidonic acid (AA) and lysophospholipids; specifically, lysophosphatidylcholines (LysoPC 20:0, 20:1 and 18:2/0:0) and 2,3-dinor-8-epi-PGF_2_α were significantly elevated, destabilizing membranes and inducing necrosis [53]. These lipid changes are mechanistically linked to the activation of ferroptosis. The marked upregulation of polyunsaturated fatty acids (PUFAs), including adrenic and arachidic acids, supplies the lipid substrates required for peroxidation in ferroptosis [54,55]. This process is further promoted by the upregulation of key ferroptosis-related enzymes—acyl-CoA synthetase long-chain family member 4 (ACSL4) and lysophosphatidylcholine acyltransferase 3 (LPCAT3)—which integrate PUFAs into membrane phospholipids, thereby facilitating peroxidation [56,57]. Ferroptosis, an iron-dependent form of cell death driven by lipid peroxidation [58,59], is also regulated by the p53–SLC7A11 axis. We observed that *slc7a11* expression was significantly downregulated in the non-cultivable (NCE) group compared to the cultivable (CE) group, while *p53* was upregulated. As *slc7a11* is a downstream target of *p53* [60,61], its suppression reduces cystine uptake, depletes glutathione, and inactivates glutathione peroxidase, thereby accelerating lipid peroxidation [58,62,63]. Additionally, mitochondrial dysfunction arising from impaired TCA cycle activity upregulates *vdac2*, enhancing ROS production and further promoting lipid peroxide formation [64,65,66]. In line with this, *vdac2* expression was significantly elevated at ND27h, reinforcing a vicious cycle of oxidative damage. In summary, post-mortem disruption of energy and lipid metabolism converges on ferroptosis as a central executioner of cell death, leading to the irreversible loss of cellular viability and in vitro culturability (Figure 8). Although ferroptosis appears to be the principal mechanism, contributions from other pathways such as autophagy and necroptosis—as suggested by KEGG enrichment results—cannot be excluded and may act in parallel or as secondary processes.

## 4. Materials and Methods

### 4.1. Experimental Animal and Sampling

All the *P. olivaceus* used in the trials were yearlings obtained from the Beidaihe Central Experiment Station of the Chinese Academy of Fishery Sciences (Approval Code: BCES2023003). The body weight and gonad weight for all *P. olivaceus* are provided in Table A9. All *P. olivaceus* were anesthetized with 100 mg/L MS222 (E808894, macklin, shanghai, China), and then the spinal column was euthanized by spinal transection, and stored at two temperatures, 19 °C (named ND group, Normal Digestion temperature) or 4 °C (named FD group, Frozen Digestion temperature), respectively. Ovarian tissue samples were collected from both ND and FD groups at ten post-mortem intervals (0, 3, 6, 9, 12, 15, 18, 21, 24, and 27 h). A portion of the collected samples was immediately processed for primary cell line establishment according to a previously published protocol [67]. The remaining tissue samples were rapidly frozen in liquid nitrogen and stored at −80 °C for subsequent metabolomic and transcriptomic analyses.

### 4.2. Detection of Cell Survival Rates

Gonadal tissues were collected from *P. olivaceus* at each time point and minced into 1–2 mm^3^ fragments. The fragments were incubated in 1 mL trypsin solution at 23 °C for 30 min with gentle agitation. Digestion was stopped by adding 1 mL of complete medium (supplemented with 10% FBS). The cell suspension was filtered through a 40 μm cell strainer, centrifuged at 300× *g* for 5 min, and the pellet was resuspended in 1 mL PBS. Cell viability was immediately assessed using the Trypan Blue Cell Viability Detection Kit (C0011, Beyotime, shanghai, China), following the manufacturer’s instructions. Approximately 500 cells per sample were counted using a hemocytometer to distinguish total cells from blue-stained (non-viable) cells. Viability (%) was calculated as [(total cells − blue cells)/total cells] × 100%. Each time point was analyzed in triplicate.

### 4.3. Alkaline Phosphatase and RT-PCR Analysis

Alkaline phosphatase staining was performed on ND15h and FD24h cells that had been passaged to the third generation. The specific procedure was as follows: cells were first washed twice with PBS, fixed with 4% paraformaldehyde for 30 min, and then subjected to alkaline phosphatase staining using the Beyotime C3250S kit (Beyotime, Shanghai, China) according to the manufacturer’s instructions. Finally, images were captured using an inverted microscope. RT-PCR primers for *vasa*, *oct4* and 18S rRNA (Table A10) were designed using Primer Premier 5.0. The reactions were performed using an S1000 thermal cycler (Bio-Rad, Hercules, CA, USA). Reactions (20 μL) contained 10 μL 2X Hieff PCR Master Mix (With Dye), 10 μM each primer, 1 μL cDNA and 8 μL RNase-free water. Cycling: 94 °C 5 min; 94 °C 30 s, 58 °C 30 s, 72 °C 15 s cycle 30 times; 72 °C 10 min. The products were resolved on a 1% agarose gel at 120 V for 20 min and imaged using a Tanon 5200 Multi system (Tanon, Shanghai, China).

### 4.4. RNA Sample Collection, Library Construction, and Sequencing

Ovarian samples from the ND and FD groups were collected in triplicate. The samples subjected to 15 h (ND15h) and 18 h (ND18h) of death at 19 °C were named ND15h_1, ND15h_2, ND15h_3, ND18h_1, ND18h_2, and ND18h_3, respectively. The samples subjected to 24 h (FD24h) or 27 h (FD27h) of death at 4 °C were named FD24h_1, FD24h_2, FD24h_3, FD27h_1, FD27h_2, and FD27h_3, respectively. Total RNA was extracted using the Total RNA Extraction Kit (DP419, TIANGEN, Beijing, China) and a Bioanalyzer 2100 system (Agilent Technologies, Santa Clara, CA, USA), and RNA integrity was evaluated using the RNA Nano 6000 Assay Kit. To create tiny fragments, polyA tail-containing mRNA was isolated using oligo (dT) magnetic beads and interrupted with divalent cations in NEB fragmentation buffer [68]. First strand cDNA was generated using random oligonucleotides as primers in an M-MuLV reverse-transcriptase device. The RNA strand was then destroyed with RNase H, and a second strand of cDNA was created using dNTPs in a DNA polymerase I apparatus. The isolated double-stranded cDNA was end-repaired, A-tailed, and ligated to sequencing junctions. cDNAs ranging from 250 to 300 bp were screened with AMPure XP beads, amplified by PCR, purified again with AMPure XP beads to create the final library, and sequenced on an Illumina NovaSeq platform (Novogene, Beijing, China).

### 4.5. Transcriptome Assembly, Functional Annotation, and Data Analysis

The raw sequencing data was filtered by fastp [69] to exclude reads with adapters, N-containing sequences, and low quality (Q ≤ 5). The error rate and GC content were evaluated to ensure clean readings were aligned to an index reference genome created using HISAT (v2.2.1) [70]. The transcriptome was assembled using StringTie (v2.2.1) [71] and annotated in databases such as Protein Family (Pfam), Gene Superfamily (superfamily), Gene Ontology Database (GO), and Kyoto Encyclopedia of Genes and Genomes (KEGG). Differential analysis was performed using DESeq2 software (v1.38.3) [72], with differential gene screening criteria of |log2 (fold change) > 1| and padj < 0.05. GO functional and KEGG pathway enrichment studies were carried out on the differential gene sets using the ClusterProfiler software (v4.6.2) [73]. A degree with a *p*-value of 0.05 was deemed statistically significant in these analyses.

### 4.6. Metabolite Extraction and LC-MS/MS Analysis

Four biological replicates of the ovarian samples from the ND and FD groups were examined using untargeted LC-MS [74,75]. A 100 mg tissue sample from each individual specimen was powdered in liquid nitrogen, added to 500 μL of 80% methanol, vortexed and agitated, stored at 4 °C for 5 min, then centrifuged at 15,000× *g* for 20 min. The supernatant was collected and diluted to a final concentration of 53% methanol with LC-MS grade water before centrifugation at 15,000× *g*, 4 °C for 20 min. Finally, the supernatant was fed into the LC-MS (Agilent 5977A, Agilent Tachnologies, Santa Clara, CA, USA) analytical system [76]. A 17 min linear gradient with a flow rate of 0.2 mL/min was used to inject the samples onto a Hypersil Gold column (25002-102130, Thermo Fisher Scientific, Waltham, MA, USA). The positive polarity mode used eluents A (0.1% FA in water) and eluent B (methanol). The eluents for the negative-polarity mode were A (5 mM ammonium acetate, pH 9.0) and B (methanol). The scanning range was set at *m*/*z* 100–1500. The data were predicted and normalized to obtain metabolite identification and relative quantification results.

### 4.7. Metabolomics Data Analysis

Statistical analyses were performed using R (v3.4.3) [77], Python (Python 2.7.6 version) [78] and CentOS (CentOS release 6.6) [79]. The identified metabolites were annotated using KEGG (accessed on 1 February 2024), HMDB (accessed on 1 February 2024), and LIPID MAPS (accessed on 1 February 2024) databases. Data were transformed for principal component analysis (PCA) and orthogonal partial least squares discriminant analysis (OPLS-DA) were performed using R (v3.4.3) with the MetaX package (source code available at https://github.com/wenbostar/metaX, accessed on 1 February 2024) [80]. The default criteria for differential metabolite screening included VIP > 1, *p*-value < 0.05, and FC ≥ 1.5 or FC ≤ 0.5. Correlation analysis (Pearson’s correlation coefficient) between differential metabolites was performed using R language cor() (v3.4.3), and statistical significance (*p*-value < 0.05) was achieved via the cor.mtest() (v0.92) in R language. The functions of these metabolites and their metabolic pathways were investigated using the Kyoto Encyclopedia of Genes and Genomes database. Metabolic pathway enrichment of differential metabolites was performed; when the ratio was satisfied by x/n > y/N, the metabolic pathway was considered to be enriched, and when *p*-value of the metabolic pathway was <0.05, the metabolic pathway was considered to be significantly enriched.

### 4.8. Combined Analysis of Transcriptomics and Metabolomics

To achieve a comprehensive understanding of the molecular mechanisms driving phenotypic divergence, we performed an extensive cross-omics analysis, integrating transcriptomic and untargeted metabolomic data. Initially, differentially expressed genes (DEGs) with |log_2_FC| ≥ 1 and a false discovery rate (FDR) < 0.05, along with differentially expressed metabolites (DEMs) characterized by |log_2_FC| ≥ 1, variable importance in projection (VIP) > 1, and *p* < 0.05, were mapped to their corresponding KEGG pathways utilizing the clusterProfiler (v4.6.2) [81] and MetaboAnalystR (v3.3) tools [82]. Pathway enrichment analysis was conducted using a hypergeometric test with an FDR threshold of <0.05. To enhance the robustness of our findings, pathways represented by fewer than three DEGs or DEMs were excluded, allowing us to focus on shared pathways for subsequent analyses.

### 4.9. qRT-PCR Validation of DEGs

We selected ten DEGs from ND group (ND18h vs. ND15h) and FD group (FD27h vs. FD24h), respectively, for qRT-PCR to verify the reliability of the RNA-Seq results. Based on the CDS of the selected gene, twenty pairs of primers were designed using Primer Premier software (version 5.0) (Table A10). *β-actin* was used as an internal reference gene. qRT-PCR mixture (20 μL) consisted of 10 μL TB Green^®^ Premix Ex Taq™ (Takara, Kusatsu, Japan), 0.5 μL of each primer (10 μM), 1 μL of cDNA, and 8 μL of RNA-free water. Reactions were performed on a q225 qRT-PCR platform (Novogen, Tianjin, China) under the following thermal cycling conditions: 94 °C for 2 min, 94 °C for 30 s, and 60 °C for 20 s. Three biological replicates of each treatment were analyzed. The relative expressions of genes were calculated using the 2^−ΔΔCt^ method [83].

## 5. Conclusions

This study establishes that viable oogonial stem cells (OSCs) can be successfully isolated and cultured from post-mortem ovarian tissues of *P. olivaceus* within defined time windows: 15 h at 19 °C and 24 h at 4 °C. Beyond these critical intervals, our predictive analysis revealed that cell culture failure is primarily attributable to ferroptosis triggered by lipid peroxidation. This mechanistic insight is further supported by the identification of key biomarkers, including the genes *vdac2*, *p53*, and *slc7a11*, and metabolites such as adrenic acid and arachidic acid.

Collectively, these findings provide both theoretical and practical foundations for genetic resource conservation. The defined post-mortem viability windows enable a strategic approach to preserve germplasm resources by establishing germline stem cell lines from unexpectedly deceased individuals in wild or captive environments. This methodology transforms previously lost genetic material into valuable biological resources, offering novel solutions for conserving endangered species and enhancing breeding programs in aquaculture. Future investigations into cryopreservation techniques and ferroptosis inhibition could further extend OSC viability, thereby substantially broadening conservation possibilities.

## Data Availability

The data are contained within the article. RNA-seq data are submitted in China National Center for Bioinformation (BioProject: CRA029868).

## Appendix A

**Table A1:** Establishment and passaging of oogonial stem cells in the ND group at different time intervals after death in *P. olivaceus*.

	ND Group	0 h	3 h	6 h	9 h	12 h	15 h	18 h
Generation								
G1		25 d	43 d	30 d	55 d	55 d	90 d	/
G2		7 d	7 d	15 d	11 d	9 d	15 d	/
G3		7 d	7 d	4 d	7 d	7 d	7 d	/

**Table A2:** Establishment and passaging of oogonial stem cells in the FD group at different time intervals after death in *P. olivaceus*.

	FD Group	3 h	6 h	9 h	12 h	15 h	18 h	21 h	24 h	27 h
Generation										
G1		45 d	32 d	18 d	17 d	36 d	30 d	21 d	36 d	/
G2		15 d	3 d	4 d	25 d	5 d	7 d	15 d	5 d	/
G3		7 d	5 d	8 d	3 d	3 d	7 d	5 d	5 d	/

**Table A3:** Comparison of transcriptome data and reference genome of *P. olivaceus* at different times chilled and room temperature deaths.

Sample	Total	Unmapped (%)	Unique Mapped (%)	Multiple Mapped (%)	Total Mapped (%)
ND15h-1	40,801,018	16,440,151 (40.29%)	37,617,362 (92.2%)	1,054,338 (2.58%)	38,671,700 (94.78%)
ND15h-2	40,310,714	16,339,041 (40.53%)	37,239,993 (92.38%)	1,103,402 (2.74%)	38,343,395 (95.12%)
ND15h-3	46,885,594	18,325,940 (39.09%)	43,250,261 (92.25%)	1,373,811 (2.93%)	44,624,072 (95.18%)
ND18h-1	38,825,814	15,930,534 (41.03%)	35,820,303 (92.26%)	1,124,155 (2.9%)	36,944,458 (95.15%)
ND18h-2	42,378,838	18,639,800 (43.98%)	37,455,412 (88.38%)	978,946 (2.31%)	38,434,358 (90.69%)
ND18h-3	37,283,930	15,355,381 (41.18%)	32,976,563 (88.45%)	931,946 (2.5%)	33,908,509 (90.95%)
FD24h-1	40,445,378	16,445,352 (40.66%)	37,346,216 (92.34%)	1,119,270 (2.77%)	38,465,486 (95.1%)
FD24h-2	48,930,460	19,085,661 (39.01%)	42,725,682 (87.32%)	1,374,441 (2.81%)	44,100,123 (90.13%)
FD24h-3	52,648,168	20,366,600 (38.68%)	46,566,537 (88.45%)	1,486,062 (2.82%)	48,052,599 (91.27%)
FD27h-1	41,534,978	17,939,371 (43.19%)	37,768,396 (90.93%)	1,139,589 (2.74%)	38,907,985 (93.68%)
FD27h-2	43,442,624	17,732,956 (40.82%)	39,693,588 (91.37%)	1,199,797 (2.76%)	40,893,385 (94.13%)
FD27h-3	43,529,080	18,317,491 (42.08%)	39,967,505 (91.82%)	1,151,657 (2.65%)	41,119,162 (94.46%)

**Table A4:** Basic information statistics of *P. olivaceus* at different times of room temperature and chilled deaths.

Sample	Raw Bases (bp)	Clean Bases (bp)	Q20 (%)	Q30 (%)	GC (%)
ND15h-1	6,400,731,900	40,801,018	39,732,031 (97.38%)	38,042,869 (93.24%)	20,849,320 (51.10%)
ND15h-2	6,585,703,200	40,310,714	39,302,946 (97.50%)	37,686,487 (93.49%)	20,526,216 (50.92%)
ND15h-3	7,423,662,900	46,885,594	45,727,520 (97.53%)	43,819,276 (93.46%)	24,061,687 (51.32%)
ND18h-1	6,238,026,600	38,825,814	37,820,225 (97.41%)	36,232,250 (93.32%)	19,719,631 (50.79%)
ND18h-2	6,912,105,300	42,378,838	41,336,319 (97.54%)	39,564,883 (93.36%)	20,841,913 (49.18%)
ND18h-3	6,019,846,500	37,283,930	36,404,029 (97.64%)	34,890,302 (93.58%)	18,656,879 (50.04%)
FD24h-1	6,327,326,700	40,445,378	39,414,021 (97.45%)	37,784,072 (93.42%)	20,675,677 (51.12%)
FD24h-2	7,364,892,300	48,930,460	47,795,273 (97.68%)	45,872,306 (93.75%)	24,797,957 (50.68%)
FD24h-3	7,921,212,600	52,648,168	51,463,584 (97.75%)	49,399,776 (93.83%)	26,724,210 (50.76%)
FD27h-1	6,653,903,100	41,534,978	40,313,850 (97.06%)	38,511,232 (92.72%)	20,796,563 (50.07%)
FD27h-2	6,899,318,700	43,442,624	42,208,853 (97.16%)	40,362,542 (92.91%)	21,968,935 (50.57%)
FD27h-3	6,795,099,300	43,529,080	42,392,971 (97.39%)	40,595,220 (93.26%)	21,847,245 (50.19%)

**Table A5:** The top 20 KEGG pathways enriched in ND15h vs. ND18h.

ID	Class	Description	*p*-Value	Q-Value	Ratio	Num	Up	Down
pov00330	Metabolism; Amino acid metabolism	Arginine and proline metabolism	0.002313977	0.141152599	0.09	3	2	1
pov00010	Glycolysis/Gluconeogenesis	Glycolysis/Gluconeogenesis	0.006390935	0.194923503	0.09	3	2	1
pov04218	Cellular senescence	Cellular senescence	0.019128015	0.304633194	0.13	4	2	2
pov00260	Glycine, serine and threonine metabolism	Glycine, serine and threonine metabolism	0.022065588	0.304633194	0.06	2	1	1
pov00620	Metabolism; Carbohydrate metabolism	Pyruvate metabolism	0.024969934	0.304633194	0.06	2	2	0
pov04217	Necroptosis	Necroptosis	0.04034051	0.35638506	0.09	3	1	2
pov00310	Metabolism; Amino acid metabolism	Lysine degradation	0.05046653	0.35638506	0.06	2	1	1
pov04020	Calcium signaling pathway	Calcium signaling pathway	0.054192134	0.35638506	0.13	4	1	3
pov00561	Metabolism; Lipid metabolism	Glycerolipid metabolism	0.055808883	0.35638506	0.06	2	2	0
pov04115	p53 signaling pathway	p53 signaling pathway	0.067057946	0.35638506	0.06	2	2	0
pov04146	Peroxisome	Peroxisome	0.074443165	0.35638506	0.06	2	0	2
pov04110	Cell cycle	Cell cycle	0.074697428	0.35638506	0.09	3	3	0
pov01230	Biosynthesis of amino acids	Biosynthesis of amino acids	0.075950914	0.35638506	0.06	2	1	1
pov00430	Biosynthesis of amino acids	Taurine and hypotaurine metabolism	0.085790241	0.359435202	0.03	1	0	1
pov01232	Nucleotide metabolism	Nucleotide metabolism	0.089950194	0.359435202	0.06	2	2	0
pov00053	Metabolism; Carbohydrate metabolism	Ascorbate and aldarate metabolism	0.095401578	0.359435202	0.03	1	1	0
pov00340	Metabolism; Amino acid metabolism	Histidine metabolism	0.100170466	0.359435202	0.03	1	1	0
pov00601	Metabolism; Amino acid metabolism	Glycosphingolipid biosynthesis—lacto and neolacto series	0.109635285	0.371541799	0.03	1	0	1
pov00982	Metabolism; Xenobiotics biodegradation and metabolism	Drug metabolism—cytochrome P450	0.128276447	0.404567961	0.03	1	0	1
pov00515	Mannose type O-glycan biosynthesis	Mannose type O-glycan biosynthesis	0.13287733	0.404567961	0.03	1	0	1

**Table A6:** The top 20 KEGG pathways enriched in FD24h vs. FD27h.

ID	Class	Description	*p*-Value	Q-Value	Ratio	Num	Up	Down
pov03010	Genetic Information Processing; Translation	Ribosome	7.00 × 10^−57^	1.06 × 10^−54^	0.092576419	106	106	0
pov00190	Metabolism; Energy metabolism	Oxidative phosphorylation	2.73 × 10^−18^	2.07 × 10^−16^	0.059388646	68	68	0
pov04260	Cardiac muscle contraction	Cardiac muscle contraction	4.21 × 10^−7^	2.13 × 10^−5^	0.036681223	42	25	17
pov03020	Genetic Information Processing; Transcription	RNA polymerase	0.000310433	0.01179644	0.013100437	15	13	2
pov03018	Genetic Information Processing; Folding, sorting and degradation	RNA degradation	0.000539594	0.016403653	0.025327511	29	19	10
pov03050	Genetic Information Processing; Folding, sorting and degradation	Proteasome	0.000994358	0.025190408	0.016593886	19	19	0
pov03420	Genetic Information Processing; Replication and repair	Nucleotide excision repair	0.005929872	0.128762935	0.017467249	20	14	6
pov03013	Genetic Information Processing; Translation	Nucleocytoplasmic transport	0.014518762	0.246774467	0.026200873	30	8	22
pov03060	Genetic Information Processing; Folding, sorting and degradation	Protein export	0.014611646	0.246774467	0.007860262	9	8	1
pov03040	Genetic Information Processing; Transcription	Spliceosome	0.02216678	0.334145793	0.031441048	36	28	8
pov03030	Genetic Information Processing; Replication and repair	DNA replication	0.024181603	0.334145793	0.011353712	13	8	5
pov04814	Cellular Processes; Cell motility	Motor proteins	0.043827537	0.555148808	0.043668122	50	15	35
pov03460	Genetic Information Processing; Replication and repair	Fanconi anemia pathway	0.104087353	1	0.012227074	14	6	8
pov03022	Genetic Information Processing; Transcription	Basal transcription factors	0.142019371	1	0.009606987	11	8	3
pov00310	Metabolism; Amino acid metabolism	Lysine degradation	0.150289521	1	0.014847162	17	4	13
pov00100	Metabolism; Lipid metabolism	Steroid biosynthesis	0.16535451	1	0.005240175	6	4	2
pov00860	Metabolism; Metabolism of cofactors and vitamins	Porphyrin metabolism	0.172198533	1	0.0069869	8	7	1
pov04110	Cellular Processes; Cell growth and death	Cell cycle	0.176882551	1	0.03580786	41	14	27
pov04150	Environmental Information Processing; Signal transduction	mTOR signaling pathway	0.184488124	1	0.033187773	38	13	25
pov04623	Organismal Systems; Immune system	Cytosolic DNA-sensing pathway	0.195926873	1	0.011353712	13	10	3

**Table A7:** The top 20 KEGG pathways enriched in FD24h and ND15h no change genes.

ID	Class	Description	*p*-Value	Q-Value	Ratio	Num
pov04080	Environmental Information Processing; Signaling molecules and interaction	Neuroactive ligand-receptor interaction	2.2803047891782 × 10^−74^	3.39765413587552 × 10^−72^	0.178278689	261
pov04060	Environmental Information Processing; Signaling molecules and interaction	Cytokine-cytokine receptor interaction	1.54860757163338 × 10^−26^	1.15371264086687 × 10^−24^	0.073770492	108
pov04020	Environmental Information Processing; Signal transduction	Calcium signaling pathway	3.69346909045443 × 10^−22^	1.83442298159237 × 10^−20^	0.099043716	145
pov04514	Environmental Information Processing; Signaling molecules and interaction	Cell adhesion molecules	2.20278383435677 × 10^−12^	8.20536978297897 × 10^−11^	0.056693989	83
pov04744	Organismal Systems; Sensory system	Phototransduction	3.28572791644228 × 10^−7^	9.791469190998 × 10^−6^	0.017759563	26
pov04672	Organismal Systems; Immune system	Intestinal immune network for IgA production	9.55222811284292 × 10^−5^	0.00220169939771074	0.012295082	18
pov04270	Organismal Systems; Circulatory system	Vascular smooth muscle contraction	0.00010343554217433	0.00220169939771074	0.035519126	52
pov04261	Organismal Systems; Circulatory system	Adrenergic signaling in cardiomyocytes	0.00013256443189031	0.00246901254395702	0.049863388	73
pov00533	Metabolism; Glycan biosynthesis and metabolism	Glycosaminoglycan biosynthesis—keratan sulfate	0.000449363736190256	0.00743946629914979	0.008196721	12
pov04350	Environmental Information Processing; Signal transduction	TGF-beta signaling pathway	0.000642067969688125	0.00956681274835306	0.030737705	45
pov00230	Metabolism; Nucleotide metabolism	Purine metabolism	0.00104623266514309	0.0141716970096655	0.035519126	52
pov04010	Environmental Information Processing; Signal transduction	MAPK signaling pathway	0.00146441859372183	0.0181831975387127	0.071721311	105
pov00140	Metabolism; Lipid metabolism	Steroid hormone biosynthesis	0.00186736245711854	0.0214028466238971	0.010245902	15
pov00604	Metabolism; Glycan biosynthesis and metabolism	Glycosphingolipid biosynthesis—ganglio series	0.00242041178360021	0.0257600968397451	0.006147541	9
pov00590	Metabolism; Lipid metabolism	Arachidonic acid metabolism	0.00817442252971953	0.0778334418749539	0.012978142	19
pov00532	Metabolism; Glycan biosynthesis and metabolism	Glycosaminoglycan biosynthesis—chondroitin sulfate/dermatan sulfate	0.00835795348992793	0.0778334418749539	0.007513661	11
pov04916	Organismal Systems; Endocrine system	Melanogenesis	0.00930495422181556	0.0815551870029717	0.028688525	42
pov04310	Environmental Information Processing; Signal transduction	Wnt signaling pathway	0.0151342015408406	0.12527755719918	0.040983607	60
pov00910	Metabolism; Energy metabolism	Nitrogen metabolism	0.0260673964408138	0.203009420682821	0.006147541	9
pov00380	Metabolism; Amino acid metabolism	Tryptophan metabolism	0.027249586668835	0.203009420682821	0.010928962	16

**Table A8:** The top 20 KEGG pathways enriched in FD27h and ND18h no change genes.

ID	Class	Description	*p*-Value	Q-Value	Ratio	Num
pov04080	Environmental Information Processing; Signaling molecules and interaction	Neuroactive ligand-receptor interaction	6.15224639748607 × 10^−59^	9.16684713225424 × 10^−57^	0.171344165	232
pov04020	Environmental Information Processing; Signal transduction	Calcium signaling pathway	5.83044313395946 × 10^−20^	4.3436801347998 × 10^−18^	0.098966027	134
pov04060	Environmental Information Processing; Signaling molecules and interaction	Cytokine-cytokine receptor interaction	2.18024245322315 × 10^−16^	1.0828537517675 × 10^−14^	0.064992614	88
pov04514	Environmental Information Processing; Signaling molecules and interaction	Cell adhesion molecules	2.28511681686016 × 10^−11^	8.51206014280408 × 10^−10^	0.056868538	77
pov04744	Organismal Systems; Sensory system	Phototransduction	6.20894376944006 × 10^−8^	1.85026524329314 × 10^−6^	0.019202363	26
pov04672	Organismal Systems; Immune system	Intestinal immune network for IgA production	6.58195396633777 × 10^−6^	0.000163451856830721	0.014032496	19
pov04010	Environmental Information Processing; Signal transduction	MAPK signaling pathway	8.10034590883145 × 10^−6^	0.000172421648630841	0.080502216	109
pov04270	Organismal Systems; Circulatory system	Vascular smooth muscle contraction	0.000236162899145753	0.00439853399658965	0.035450517	48
pov00230	Metabolism; Nucleotide metabolism	Purine metabolism	0.00188063675192268	0.0311349862262755	0.035450517	48
pov04261	Organismal Systems; Circulatory system	Adrenergic signaling in cardiomyocytes	0.0030049955859055	0.044774434229992	0.046528804	63
pov00910	Metabolism; Energy metabolism	Nitrogen metabolism	0.00446926225877797	0.0605381887779925	0.007385524	10
pov00604	Metabolism; Glycan biosynthesis and metabolism	Glycosphingolipid biosynthesis—ganglio series	0.0066887673854356	0.0795442582859311	0.005908419	8
pov04916	Organismal Systems; Endocrine system	Melanogenesis	0.00694010307192687	0.0795442582859311	0.029542097	40
pov04350	Environmental Information Processing; Signal transduction	TGF-beta signaling pathway	0.0102746435496404	0.109351563492602	0.028064993	38
pov04810	Cellular Processes; Cell motility	Regulation of actin cytoskeleton	0.0125372806301676	0.124536987592998	0.052437223	71
pov00532	Metabolism; Glycan biosynthesis and metabolism	Glycosaminoglycan biosynthesis—chondroitin sulfate/dermatan sulfate	0.0147359052616125	0.137228117748767	0.007385524	10
pov00533	Metabolism; Glycan biosynthesis and metabolism	Glycosaminoglycan biosynthesis—keratan sulfate	0.0158685919402005	0.139083541122934	0.006646972	9
pov00561	Metabolism; Lipid metabolism	Glycerolipid metabolism	0.0256715684407054	0.212503538759172	0.017725258	24
pov04814	Cellular Processes; Cell motility	Motor proteins	0.0301713061938436	0.236606559099089	0.043574594	59
pov04912	Organismal Systems; Endocrine system	GnRH signaling pathway	0.0320475961612918	0.238754591401624	0.025110783	34

**Table A9:** Basic information of all *P. olivaceus* used in the experiment.

Group (*n* = 3)	Body Length (cm)	Overall Length (cm)	Body Weight (g)	Gonad Weight (g)
0h	16.67 ± 0.29	26.00 ± 0.00	226.96 ± 36.13	1.23 ± 0.46
ND3h	13.00 ± 1.00	21.90 ± 1.49	140.07 ± 38.12	0.25 ± 0.03
ND6h	14.60 ± 0.85	19.67 ± 0.58	121.17 ± 9.02	0.21 ± 0.04
ND9h	11.83 ± 1.04	19.33 ± 0.76	135.04 ± 27.57	0.22 ± 0.03
ND12h	15.23 ± 0.64	23.67 ± 1.15	158.22 ± 32.66	0.25 ± 0.06
ND15h	15.83 ± 0.58	20.83 ± 1.44	112.22 ± 10.53	0.27 ± 0.07
ND18h	16.00 ± 1.73	25.50 ± 2.29	223.36 ± 34.30	0.80 ± 0.12
FD3h	13.83 ± 1.26	22.50 ± 1.32	163.70 ± 34.14	0.24 ± 0.04
FD6h	12.67 ± 0.58	21.00 ± 1.73	118.42 ± 11.46	0.23 ± 0.02
FD9h	14.00 ± 1.00	21.67 ± 1.26	143.88 ± 49.04	0.25 ± 0.04
FD12h	10.83 ± 1.04	18.50 ± 1.00	130.89 ± 26.28	0.24 ± 0.02
FD15h	13.27 ± 0.40	20.93 ± 0.12	143.05 ± 5.14	0.25 ± 0.04
FD18h	12.67 ± 0.29	20.50 ± 1.32	151.41 ± 42.16	0.39 ± 0.15
FD21h	13.00 ± 1.00	23.33 ± 0.58	167.30 ± 38.56	0.55 ± 0.17
FD24h	14.83 ± 1.76	24.83 ± 2.02	211.88 ± 7.61	0.68 ± 0.17
FD27h	13.33 ± 1.04	22.83 ± 0.76	201.65 ± 8.40	0.69 ± 0.35

**Table A10:** Primer sequence table.

NCBI Reference Sequence	Forward	Reverse	Gene Name	Usage
XM_069535616.1	GCCTGACTCCAGCACA	CCATCGCCTACCCAA	*trmt61b*	qRT-PCR
XM_069530630.1	GGCTGAGGATGAGGTG	GGCTGGTGGCTTGTAG	*loc109627311*	
XM_020103989.1	TCACGGGCCATACCAG	TGACCGAGGCAGAGCA	*loc109640143*	
XM_020081586.1	CTAACCCACGGCTTTGT	GCTGCGGATTTGAACC	*loc109625962*	
XM_020086501.1	TTGGCAAACTCGGGAC	GCAGCGTTGAGGAAAG	*loc109629024*	
XM_020085544.2	CACGACTCCCAGACCCA	GCAAGATGCCGACCA	*tmprss2*	
XM_020104037.2	CGGCGGGCTTCATTT	AGAACGGCGTGGAGGT	*loc109640177*	
XM_069511009.1	TGCCTACGCTGGAGT	CGAGAAGAAATAGTGCC	*dhrs1*	
XM_020085444.1	GGTTGATGGCATTTAGGT	CTGCTGTTTACAAGGCTCT	*loc109628360*	
XM_020085379.1	AAGCACCACATTCTTCCATA	AGCCACGCTTCCTTTG	*tmlhe*	
XM_020108792.2	TGGGCTTGGGACATT	GGAAGGCGTGATTGC	*rbx1*	
XM_020106609.2	AGGACAAAGAGGGAATC	CAGCACCAAGTGAAGG	*rps27a*	
XM_020096236.2	GGCGGTTCAAGGGTCA	TGGGCGATCTCAGCAC	*rpl32*	
XM_020098086.2	GGAACATAGTGGAGGCT	ATCTGGGTGGTGGAGTA	*mrps26*	
XM_020094857.2	CAGCAACACCTCCTCAG	ACAAGCGTCCATCACC	*rnf7*	
XM_020112725.2	CGAGCGGAGAAATGA	CCAGCCGATGATGAC	*eif3f*	
XM_020086721.1	CAAGGCTGTGGTGAGGT	TCGTCTGGCGAGTTCTAT	*nudt2*	
XM_020089424.1	GAGTGCGACAAGAAGGAT	GGAGGAGCAGATTAGGG	*loc109630913*	
XM_020108373.1	TCCGCACCTCTTCCAC	TTTTCAGCCAGTCGTTC	*ntpcr*	
XM_020106718.1	CAGGTCCCTCAGGTTGT	AGGCTGCTCTGCTTCA	*hddc3*	
XM_069524822.1	GGAAATCGTGCGTGACATTAAG	CCTCTGGACAACGGAACCTCT	*β-actin*	
JQ070418.1	CTAACCACGAGGGAACTA	GCTGGATGAGGCTGAC	*vasa*	RT-PCR
KJ522774.2	AGACTTTCTTCCCATTCCC	TGACGGTGTCAGATACTGTTG	*oct4*	
XR_011242831.1	CCTGAGAAACGGCTACCACAT	ATCCCGAGGTCCAACTACGAG	18s rRNA	
